# Impact of an Electron Wigner Crystal on Exciton Propagation

**DOI:** 10.1021/acs.nanolett.5c06312

**Published:** 2026-03-11

**Authors:** Daniel Erkensten, Alexey Chernikov, Ermin Malic

**Affiliations:** † Department of Physics, 9377 Philipps-Universität Marburg, 35037 Marburg, Germany; ‡ mar.quest—Marburg Center for Quantum Materials and Sustainable Technologies, 35032 Marburg, Germany; § Institute of Applied Physics and Würzburg−Dresden Cluster of Excellence ct.qmat, TU Dresden, 01187 Dresden, Germany

**Keywords:** exciton propagation, Wigner crystal, transition-metal dichalcogenides, 2D materials

## Abstract

The strong Coulomb interaction in 2D materials facilitates the formation of tightly bound excitons and charge-ordered phases of matter. A prominent example is the formation of a crystalline phase from free charges due to Coulomb repulsion, known as the Wigner crystal. While exciton–electron interactions have been used as a sensor for Wigner crystallization, its impact on the exciton properties has so far been poorly understood. Here, we show that the weak potential induced by periodically ordered Wigner crystal electrons has a major impact on exciton propagation, albeit having only a minor influence on exciton energy. The effect is tunable with carrier density determining the Wigner crystal confinement and temperature via thermal occupation of higher subbands. Our work provides microscopic insights into the interplay between excitons and charge-ordered states identifying key signatures in exciton transport and establishes a theoretical framework for understanding exciton propagation in the presence of strong electronic correlations.

## Graphical abstract

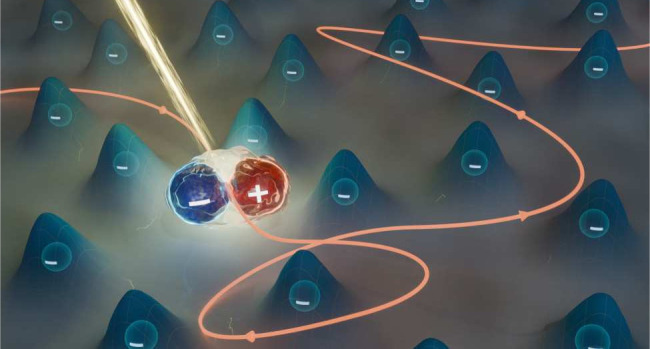

In recent years, transition-metal dichalcogenides (TMDs) have been shown to offer an exceptional material platform to study rich exciton phenomena^1–5^ and strongly correlated states of matter.^6–10^ The remarkably strong Coulomb interaction in these materials facilitates the formation of tightly bound excitons that are stable even at room temperature, as well as higher-order charge complexes, such as trions,^11–14^ biexcitons,^15–17^ Wigner crystals,^6,18,19^ or Mott insulating states in TMD-based heterostructures.^7–9,20^ The century-old prediction that an electron gas forms a triangular crystal at sufficiently low carrier densities and temperatures,^21^ resulting in a Wigner crystal (Figure 1), has recently been verified by optical spectroscopy on a two-dimensional solid-state platform in MoSe_2_ ^6,19,22^ and WSe_2_ monolayers.^23,24^ Despite these advances, little attention has been devoted to understanding the complex interplay between optically excited electron–hole pairs and electrons confined to a Wigner crystal lattice in atomically thin semiconductors. In particular, the understanding of the impact of correlated states on exciton propagation and dynamics remains lacking.

**Figure 1:**
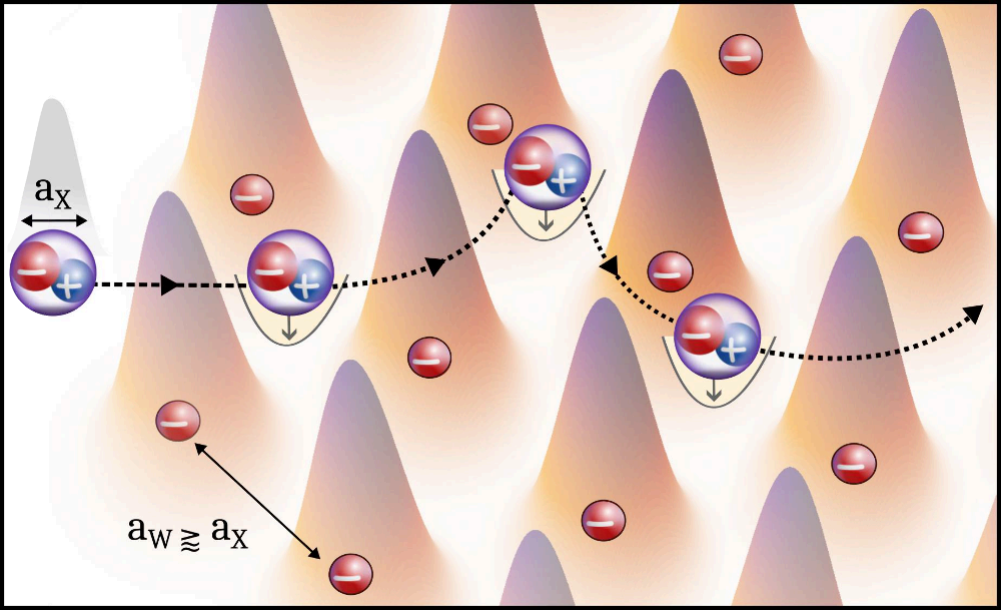
Schematic illustration of exciton transport in the presence of an electron Wigner crystal. The periodic arrangement of electrons in a Wigner crystal lattice gives rise to a potential that excitons can be trapped in, slowing down their propagation. The colored Gaussian curves illustrate the real-space charge density of electrons confined in a Wigner lattice with the periodicity set by the Wigner lattice constant *a*_W_ that is generally comparable to the spatial extent of the exciton *a*_X_.

Only recently, exciton propagation experiments in the vicinity of correlated states were reported using externally defined periodic potentials in moiré heterostructures, where the charge trapping is determined by the spatial variation of atomic registries.^25–27^ In particular, exciton propagation has been shown to be suppressed in the presence of generalized Wigner crystal states due to effective exciton–electron scattering,^28^ whereas propagation in the vicinity of Mott insulating states has been shown to be complex and could be enhanced due to strong exciton-electron repulsion in mixed exciton–electron lattices.^26^ The long-range dipolar repulsion between moiré excitons could also freeze the propagation of excitonic Mott insulating states.^27^ Furthermore, in a recent *ab initio* study,^30^ moiré exciton states are found to show substantial changes of their wave functions by generalized Wigner crystal states. In the absence of external potentials, exciton diffusion has been studied in the presence of a Fermi sea of *free* carriers demonstrating a nonmonotonous dependence with respect to the carrier density. This was explained by considering two distinct regimes governed by elastic exciton–electron scattering and trion formation, respectively.^31^ However, the impact of a spontaneous symmetry breaking in the distribution of free charges forming a Wigner crystal on exciton transport has remained a major open question.

In this work, we introduce a bridge between the physics of electronic correlations and the field of exciton transport. In particular, we investigate exciton diffusion in the presence of an electronic Wigner crystal based on a microscopic theory that could be generalized to any type of charge-ordered states including Mott insulating and generalized Wigner crystal states. We consider the exemplary case of a MoSe_2_ monolayer, which offers an ideal platform that hosts low-energy bright excitons and where the formation of electron Wigner crystal states has been experimentally verified.^6^ Despite the fact that the exciton–electron interaction is weak leading to only tiny exciton energy shifts in the submillielectronvolt range,^6,32^ we predict a significant flattening of exciton bands and a 2-fold decrease in the exciton diffusion coefficient under experimentally realistic conditions for Wigner crystallization. As excitons become subject to a periodic potential induced by the periodically arranged Wigner electrons (Figure 1), they feel the shallow potential from Wigner electrons slowing down their propagation, in analogy to exciton trapping in moiré potential pockets in twisted or lattice-mismatched TMD heterostructures.^33–36^ Here, however, the strong impact of the correlated states on exciton properties is a consequence of the Coulomb interaction alone. Interestingly, we find that exciton diffusion becomes more efficient at higher carrier densities at cryogenic temperatures, in direct contrast to what is expected from excitons scattering with free electrons as shown in recent transport experiments on doped monolayer TMDs.^31^ Altogether, our work demonstrates substantial impact of Wigner crystal formation on exciton transport that should be accessible at realistic experimental conditions.

To microscopically model exciton propagation in the presence of an electronic Wigner crystal, we start from the many-particle exciton Hamiltonian

1 $$H=\underset{Q}{\sum }E_{Q}X_{Q}^{†}X_{Q}+\underset{q,Q}{\sum }V_{\mathrm{x-e}}\left(q\right)\;\rho_{e}\left(q\right)\;X_{Q+q}^{†}X_{Q}$$

with $X_{Q}^{†}$ being bosonic annihilation (creation) exciton operators. The first term contains the free parabolic center-of-mass exciton dispersion $E_{Q}=\frac{\hbar^{2}|Q{|}^{2}}{2M}$ with **Q** being the center-of-mass momentum and $M=m_{e}^{*}+m_{h}^{*}$ the total exciton mass with $m_{e}^{*}$ and $m_{h}^{*}$ as electron and hole masses, respectively. The second term is of key importance and describes the mean-field interaction of excitons with Wigner crystal electrons, where the exciton–electron interaction *V*_x–e_(**q**) is weighted by the Wigner electron momentum charge density ρ_e_(**q**). Throughout this work, the Wigner crystal is hence treated within a symmetry-broken effective description, in which the electron density is replaced by a static, spatially periodic expectation value representing localized electrons. This does not imply a breaking of translational symmetry at the level of the exact Wigner crystal ground state, but rather constitutes a controlled mean-field description appropriate in the presence of a weak pinning.^37,38^ Furthermore, we assume a low density of excitons (*n*_x_ < *n*_e_) such that excitons act as a dilute probe of the static Wigner crystal background and the back-action of the excitons on the Wigner crystal lattice can be neglected.

The exciton–electron interaction is approximated by a contact-like and doping-independent interaction in real space,^39–41^ such that *V*_x–e_(**q**) = *v*_x–e_, with *v*_x–e_ being the long-wavelength limit of the exciton–electron interaction^39^ (see Section SI for details). The Wigner electron momentum density ρ_e_ is assumed to have a Gaussian density profile in momentum and real space, that is, $\rho_{e}\left(r\right)=\frac{1}{2\pi \xi^{2}}\underset{n}{\sum }e^{-|r-R_{n}{|}^{2}/\xi^{2}}$ with **R**_*n*_ being real-space Wigner lattice vectors and ξ describing the spatial extension of the electron wave function around the lattice sites. The latter is defined as a fraction of the Wigner lattice period (set by the carrier density) and is obtained from a variational approach by minimizing the Hartree–Coulomb repulsion energy and the kinetic energy of carriers^42^ (see Section SII).

The many-particle Hamilton operator in eq 1 describes excitons in the presence of an effective periodic potential induced by Wigner crystal electrons (Figure 1). As such, the Wigner crystal potential is expected to renormalize the free parabolic exciton band structure. It can result in a density-dependent flattening of exciton bands even in the absence of static external lattice-induced potentials.^43^ In particular, the energy spectrum of the Hamiltonian is now given by a series of subbands η defined in the mini-Brillouin zone (mBZ) spanned by the reciprocal Wigner lattice vectors (Section SIII). By exploiting the periodicity of the Wigner crystal potential and zone-folding, the Hamiltonian in eq 1 is diagonalized for the exemplary case of an hBN-encapsulated MoSe_2_ monolayer. The resulting renormalized exciton band structure is displayed in Figure 2 along a horizontal cut of the Wigner mBZ and shown for the two different electron densities of *n*_e_ = 10^11^ cm^–2^ (Figure 2a) and *n*_e_ = 10^12^ cm^–2^ (Figure 2b), corresponding to the Fermi energies $E_{F}=\hbar^{2}\pi n_{e}/m_{e}^{*}$ of 0.5 up to 5 meV, respectively. Importantly, we note that the higher-lying excitonic resonances can be understood as umklapp processes, where the energy of the umklapp-scattered excitons is provided by the reciprocal Wigner lattice vector.^6,32^ Concretely, we obtain a splitting Δ*E* ≈ 0.5 meV between the first umklapp-scattered exciton state η = 1 and the ground state η = 0 at *n*_e_ = 10^11^ cm^–2^, which grows linearly with the increasing carrier density. This can be well understood in the limit of a weak exciton–electron interaction, where the energy splitting is determined by the exciton kinetic energy at the momentum corresponding to the magnitude of a reciprocal Wigner lattice vector resulting in $\Delta E=\frac{\hbar^{2}|G_{W}{|}^{2}}{2M}∼a_{W}^{-2}∼n_{e}$ .^6,32^ Here, **G**_W_ is the (first-shell) reciprocal Wigner lattice vector with $\left|G_{W}\right|\propto a_{W}^{-1}$ , where *a*_W_ is the Wigner lattice period. The obtained splitting is in good agreement with the umklapp resonance energy measured previously.^6^

**Figure 2:**
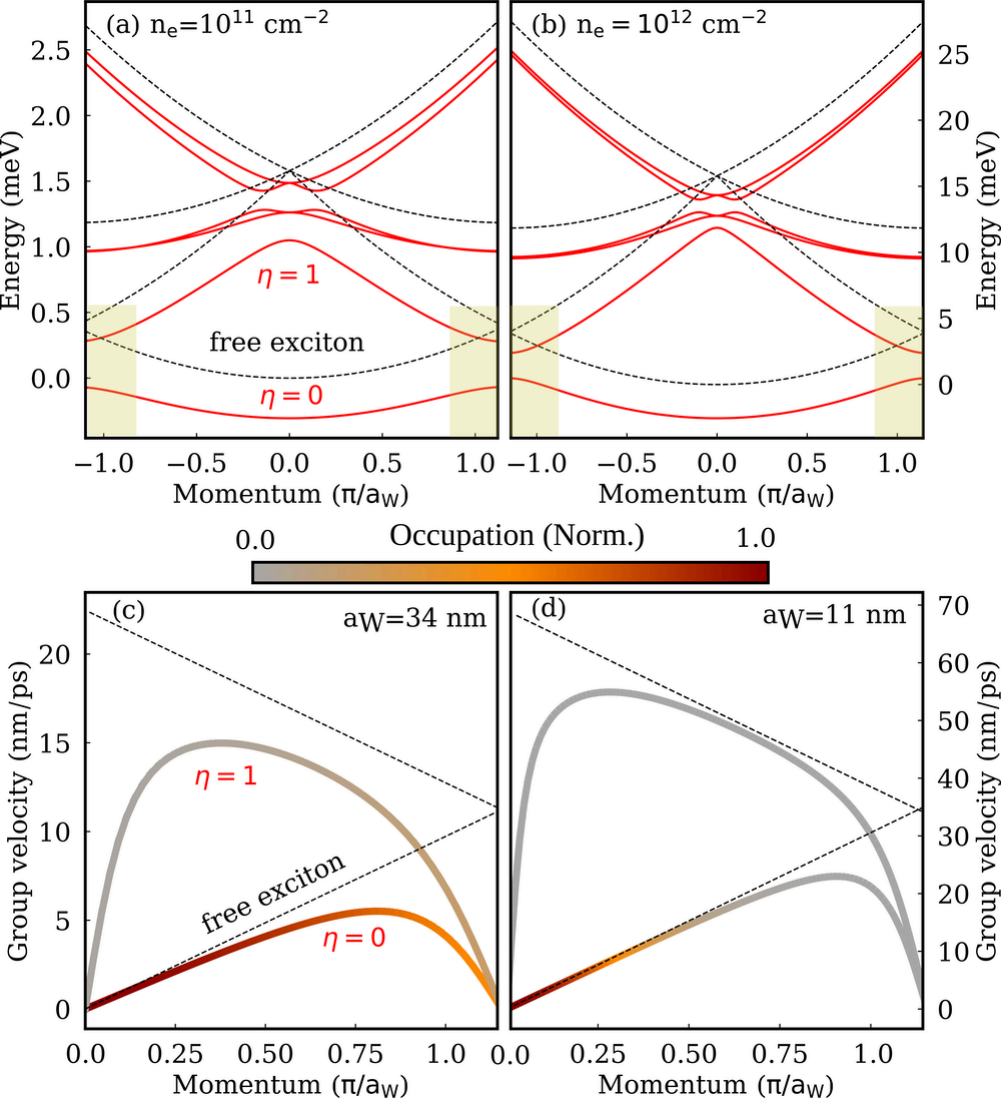
Exciton band structures and group velocities along a horizontal cut of the Wigner crystal Brillouin zone in hBN-encapsulated MoSe_2_ monolayers for (a) low carrier density *n*_e_ = 10^11^ cm^–2^ (corresponding to a Wigner lattice period *a*_W_ ≈ 34 nm) and (b) high carrier density *n*_e_ = 10^12^ cm^–2^ (*a*_W_ ≈ 11 nm). The bare bands without a Wigner crystal potential are shown with dashed lines. The flattening of the bands at the edges of the Brillouin zone (yellow areas in [(a)-(b)]), results in quenched group velocities at larger momenta [(c)-(d)]. The group velocities are superimposed by the corresponding exciton occupations for the two lowest-lying bands.

We find a generally small energy renormalization of exciton energy in the presence of a Wigner crystal potential due to the weak exciton–electron interaction; cf. the solid red and dashed black lines in Figure 2a,b denoting the renormalized and bare exciton bands, respectively. Interestingly, we show that exciton bands become flattened around the edges of the Brillouin zone for the lowest-lying η = 0 subband (yellow areas in Figure 2a,b). The band flattening is more pronounced at low densities, which is traced back to the stronger localization of Wigner crystal electrons at low densities, i.e., the spatial extent ξ of Wigner electrons is much smaller than the Wigner period *a*_W_. In particular, we find that the Lindemann ratio ξ/*a*_W_ scales with ${a_{W}}^{-1/4}∼{n_{e}}^{1/8}$ .^42^ The delocalization of Wigner electrons at elevated carrier densities also results in delocalized exciton wave functions, and thereby in more disperse bands. We show this by performing a harmonic approximation of the exciton–electron interaction potential around its minima, such that the Wannier function associated with the lowest-lying exciton subband becomes a Gaussian with the width *a*_x_ ∝ ξ (see Section SIV and Figure 1). Because $\xi /a_{W}\propto {n_{e}}^{1/8}$ , we find that excitons are more localized at lower electron densities and we expect the lowest-lying exciton subband to become more disperse as *n*_e_ increases.

The flattening of exciton bands at finite momenta results in suppressed group velocities $v_{Q}^{\eta }=\frac{1}{\hbar }\left|\nabla_{Q}\epsilon_{Q}^{\eta }\right|$ compared to the free exciton case with $v_{Q}=\frac{\hbar Q}{M}$ ; cf. Figure 2c,d. Due to the small size of the Brillouin zone at the lower density *n*_e_ = 10^11^ cm^–2^ (*a*_W_ ≈ 34 nm), the flat parts of the band structure exhibiting small group velocities are strongly occupied even at cryogenic temperatures, as shown in Figure 2c,d, where a thermal Boltzmann distribution is mapped on the momentum-dependent group velocities. Since exciton diffusion depends not only on the group velocity, but also on the occupation of exciton states (cf. eq 2), it is expected that the occupied flat parts contribute significantly to exciton transport. In contrast, at higher densities, only a small part of the states in the much larger Brillouin zone is populated (*a*_W_ ≈ 11 nm); i.e., the flat regions at higher momenta remain unoccupied (Figure 2c) and do not contribute to exciton diffusion. As a result, considering the changes in the group velocity and subband occupation, exciton diffusion is expected to be slowed down at lower carrier densities.

To obtain microscopic access to exciton propagation in the vicinity of correlated states, we calculate the exciton diffusion coefficient *D*. The latter is derived using the Wigner function formalism^44^ and by applying the relaxation-time approximation yielding^44–47^

2 $$D=\frac{1}{2}\underset{\eta }{\sum }\int_{mBZ}d^{2}Q\;\tau_{Q}^{\eta }{\left(v_{Q}^{\eta }\right)}^{2}N_{Q}^{\eta }$$

where $\tau_{Q}^{\eta }$ describes the relaxation time due to exciton–phonon scattering. Furthermore, $v_{Q}^{\eta }=\frac{1}{\hbar }\left|\nabla_{Q}\epsilon_{Q}^{\eta }\right|$ corresponds to the band-specific group velocity obtained directly from the renormalized exciton band structure $\epsilon_{Q}^{\eta }$ . Moreover, the exciton occupation $N_{Q}^{\eta }$ is estimated by a Boltzmann distribution. To derive eq 2, we perform a zone-folding of the exciton dispersion into the mBZ of the Wigner lattice. Importantly, considering low temperatures, such that only the lowest exciton subband is occupied, and assuming that the band is parabolic and $\tau_{Q}^{\eta }\approx \tau$ , the diffusion coefficient reduces to the well-known semiclassical expression $D\approx \frac{k_{B}T\tau }{M}$ with *T* as the temperature of the excitonic system.^48^ The relaxation time $\tau_{Q}^{\eta }$ is obtained from microscopically calculated exciton–phonon scattering rates explicitly taking into account the superlattice potential given by the periodic potential induced by the Wigner crystal electrons (Section V).^49^ Because exciton–electron interaction is weak and exciton–phonon scattering quickly thermalizes excitons,^50^ the applied relaxation-time approximation is well justified. We note that the semiclassical approximation of exciton transport $\left(k_{B}T\gg \frac{\hbar }{\tau }\right)$ is expected to hold at the considered cryogenic temperatures and that quantum corrections become important at higher temperatures.^48,51^ Furthermore, we remark on the exciton transport being diffusion-driven rather than hopping-driven due to the exciton–electron potential energy being similar to the thermal energy resulting in similar length scales for the localization of excitons and the Wigner lattice period (Figure 1 and Section SIV). The material-specific input parameters used for transport calculations including electron and hole masses, dielectric constants, electron–phonon coupling strength, and phonon energies, are extracted from *ab initio* calculations^52,53^ and provided in Section SVII.

We now evaluate the exciton diffusion coefficient (eq 2) for the exemplary case of a bright (KK) exciton in a MoSe_2_ monolayer. In Figure 3, the diffusion coefficient is shown as a function of the Wigner electron density. Considering free exciton propagation, we find that the exciton diffusion coefficient is density independent (blue line in Figure 3) and is given by *D* ≈ 1 cm^2^/s. Taking into account the impact of a Wigner crystal potential on the propagation of excitons, we reveal an intriguing drop in the exciton diffusion coefficient at low carrier densities down to *D* ≈ 0.5 cm^2^/s at the lowest considered density of *n*_e_ = 10^11^ cm^–2^ (red line in Figure 3). This characteristic behavior has the opposite density dependence compared to the diffusion obtained for excitons scattering with free electrons (dashed gray line in Figure 3). The latter case cannot be directly treated with a theoretical approach that relies on excitons interacting with a static potential, but is instead obtained within a Fermi-polaron approach further discussed in Section VI.

**Figure 3:**
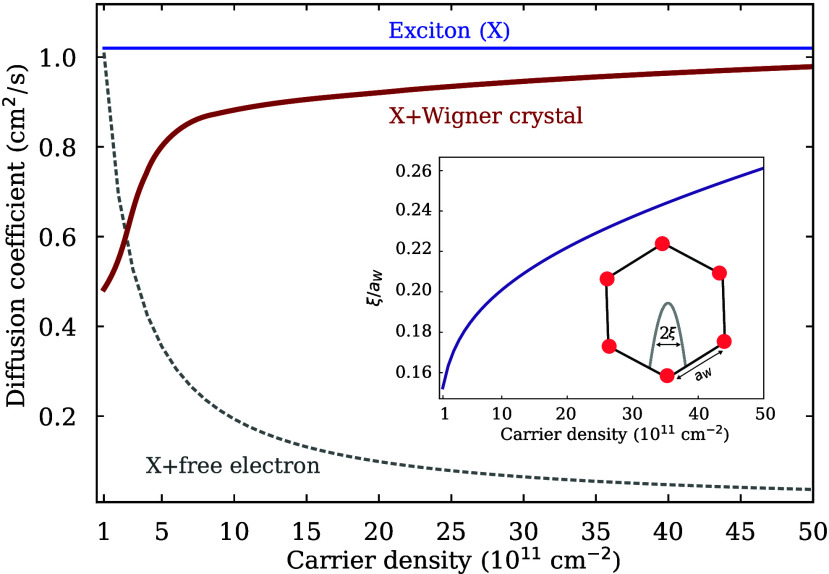
Density-dependent exciton diffusion coefficient in the presence of an electron Wigner crystal in hBN-encapsulated MoSe_2_ monolayers at cryogenic temperatures (*T* = 4 K). At low carrier densities, the Wigner crystal electrons are strongly localized (inset), i.e., their spatial extent ξ is much smaller than the Wigner lattice period *a*_W_. This gives rise to the emergence of flattened exciton bands and suppressed exciton diffusion. As the carrier density is increased, Wigner electrons become less confined, exciton bands become increasingly parabolic, and exciton diffusion approaches the limit of free excitons (blue line). In strong contrast, free electron–exciton scattering gives rise to a decrease in the exciton diffusion as a functon of the carrier density (dashed gray line).

To obtain a better understanding of the predicted density dependence of the diffusion coefficient in the presence of a Wigner crystal, we come back to eq 2. Here, the diffusion coefficient is determined by the exciton group velocity, exciton occupation, and exciton–phonon scattering time. The latter is enhanced at lower densities reflecting the decrease in the number of scattering channels when exciton bands are flattened, and therefore a boost in exciton transport at lower carrier densities would be expected, in contrast to the behavior shown in Figure 3. We find that the predominant density dependence of the diffusion coefficient can be traced back to the suppression of group velocities and the occupation of flat parts in the excitonic band structure (Figure 2c). At low carrier densities, the Wigner crystal electrons are well-localized, i.e., their spatial extent ξ is small compared to the Wigner lattice constant (inset in Figure 3). The degree of localization is described by the Lindemann parameter ξ/*a*_W_, which is growing with increasing density: the smaller the Lindemann parameter, the more localized Wigner electrons and the flatter exciton bands (Figure 2a). Upon increasing the carrier density, Wigner crystal electrons start to overlap and become delocalized (consistent with the Wigner crystal approaching quantum melting). As a consequence, excitons become more mobile and their bands more parabolic. In addition, the size of the Brillouin zone increases with density such that the flatter parts of the band structure are no longer populated (Figure 2b). This eventually leads to a diffusion coefficient that corresponds to the case of free excitons (blue line in Figure 3).

For a comparison with experimentally realistic scenarios, it is important to note that while Wigner crystal melting is not included in the microscopic model, it is expected to take place only in the upper range of the considered density regime (*n*_e_ ≳ 5 × 10^11^ cm^–2^).^6,54^ We thus expect our findings to hold and be experimentally accessible for the considered free carrier densities, predicting a drastic drop in the diffusion coefficient in the low-density limit. The predicted, characteristic density dependence of the exciton diffusion coefficient in Figure 3 strongly contrasts the density dependence of exciton diffusion for the case of a Fermi sea of free electrons. Applying a Fermi-polaron approach, we indeed find a rapid decrease in exciton diffusion as a function of the carrier density instead. This reflects the efficient scattering between excitons and free electrons.^31^ The decrease in exciton diffusion is a qualitatively different behavior compared to the predicted exciton propagation in the presence of a Wigner crystal, where we find a monotonous increase in the diffusion coefficient as a function of the carrier density. Thus, the drop in the exciton diffusion at low densities (red line in Figure 3) is identified as a clear hallmark for the Wigner crystallization.

Now, we study the density dependence of the exciton diffusion coefficient at different temperatures. At *T* = 4 K, excitons predominantly occupy only the energetically lowest subband (Figure 2c,d) even at low densities, where the separation between different subbands is very small. At elevated temperatures, also higher-lying bands (η ≥ 1) become populated, as shown in Figure 4a for the case of *T* = 12 K and *n*_e_ = 10^11^ cm^–2^. These bands are more disperse and have higher group velocities. As a consequence, for a fixed low density, the diffusion coefficient is expected to be larger at elevated temperatures. Furthermore, at higher densities (*n*_e_ = 10^12^ cm^–2^), we find that only the first exciton subband is occupied at *T* = 12 K (Figure 4b), but a larger part of the Brillouin zone is populated compared to the case of *T* = 4 K (Figure 2d).

**Figure 4:**
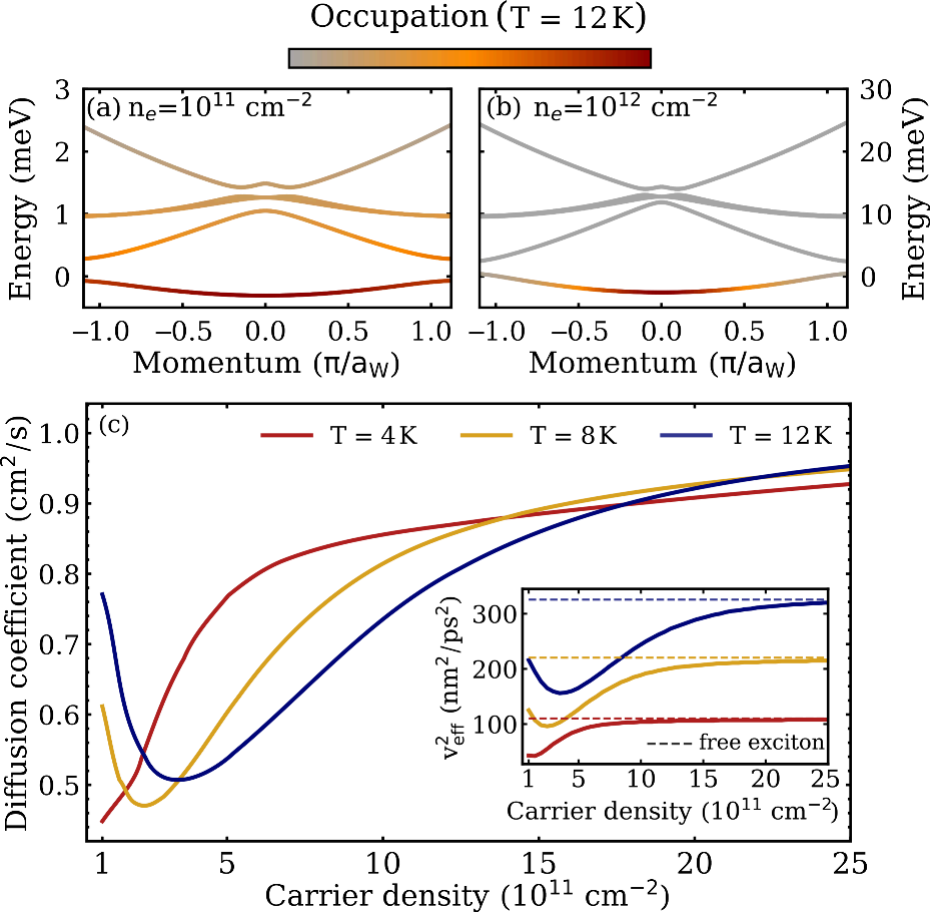
Temperature-dependent exciton diffusion in the presence of a Wigner crystal. Exciton band structure with the bands overlaid by the exciton occupation at *T* = 12 K, revealing (a) a large occupation of higher-lying exciton bands at low densities and (b) their negligible occupation at high densities. (c) Exciton diffusion coefficient at three different temperatures. For *T* = 8 and 12 K, exciton diffusion becomes nonmonotonous as a function of the carrier density, reflecting the behavior of the effective thermal group velocity (inset).

In Figure 4c, we show the density-dependent exciton diffusion coefficient for three different temperatures of *T* = 4, 8, and 12 K. Note that for *T* > 12 K, the Wigner crystal has been shown to melt.^6^ For higher temperatures considered here (*T* = 8 K and *T* = 12 K, yellow and blue lines, respectively), we again find a drop in the exciton diffusion coefficient, when the density is reduced. However, in contrast to the case of *T* = 4 K discussed so far, the diffusion coefficient becomes nonmonotonous and increases again for very low densities. Thus, the diffusion coefficient exhibits a minimum at a specific temperature-dependent carrier density. This can be directly traced back to density-dependent minima found in the squared effective group velocity (thermally averaged velocity), $v_{\mathrm{eff}}^{2}=\underset{\eta ,Q}{\sum }{\left(v_{Q}^{\eta }\right)}^{2}N_{Q}^{\eta }$ ; see the inset in Figure 4c. At low carrier densities, several subbands with large group velocities are occupied (Figure 4a). As the density increases, the energy separation between the subbands becomes larger, such that only the lowest-lying band is eventually populated (Figure 4b), leading to a decrease in the squared effective group velocity. The higher the density, the more parabolic the relevant subband becomes, leading to an increase in the squared effective group velocity. In the limit of high carrier densities, the thermally averaged squared group velocity approaches $v_{\mathrm{eff}}^{2}=\frac{2k_{B}T}{M}$ , as expected from the equipartition theorem (dashed lines in the inset in Figure 4c). Here, the diffusion coefficient approaches the value of free excitons. Overall, the density-dependent subband separation, dispersion, and thermal occupation determine the diffusion coefficient.

In summary, we have developed a microscopic many-particle approach to describe exciton transport in the presence of an electronic Wigner crystal in atomically thin semiconductors. We show that in spite of a weak Wigner potential giving rise to only small energy shifts, it has a substantial impact on exciton transport. Considering the exemplary case of hBN-encapsulated MoSe_2_ monolayers, we predict the diffusion coefficients to be substantially decreased at low carrier densities. This behavior is explained by a partial trapping of excitons in the periodic Wigner crystal potential that is reflected by a flattening of exciton subbands. We also show that exciton diffusion is both strongly density- and temperature-dependent when excitons interact with Wigner crystal electrons. This opens up pathways for theoretical understanding of exciton transport in the presence of electronic correlations, predicting both strong and characteristic density-dependent effects, to be extended for a variety of scenarios introducing the physics of strongly correlated electrons to the field of exciton transport.

## Supplementary Material



## Notes

Detailed derivation of the exciton–electron interaction and details on real-space exciton wave functions, exciton–phonon scattering rates, and input parameters (PDF)
