# Supplementary material for: Impact of an Electron Wigner Crystal on Exciton Propagation
Source: Nano Lett. 2026 Mar 11;26(15):5002–8. doi: 10.1021/acs.nanolett.5c06312 (PMC13107519; doi:10.1021/acs.nanolett.5c06312)
Supplement: Supplementary file 1 [file nl5c06312_si_001.pdf]

Supplemental Material
Impact of an electron Wigner crystal on exciton propagation

Daniel Erkensten[1,2], Alexey Chernikov[3] and Ermin Malic[1,2]
[1] *Department of Physics, Philipps-Universität Marburg, 35032 Marburg, Germany*
[2] *mar.quest—Marburg Center for Quantum Materials and Sustainable Technologies, 35032 Marburg, Germany*
[3] *Institute of Applied Physics and Würzburg-Dresden Cluster of Excellence ct.qmat, TU Dresden, 01187 Dresden, Germany*

I. EXCITON-ELECTRON INTERACTION

We consider the local exciton-electron interaction $V_{x-e}(\mathbf{q})$ in the limit of low doping [1]

$$V_{x-e}(\mathbf{q}) = \sum_{\mathbf{k},\nu\neq\mathbf{0}} \frac{\Lambda_{\mathbf{k}}^{0\nu}\Lambda_{\mathbf{q-k}}^{\nu 0}}{E_0^X - E_\nu^X} \ , \tag{S1}$$

with the exciton energies E_ν^X and $\nu = (n, l)$, where n and l are the principal quantum number and the magnetic quantum number, respectively. Furthermore, the scattering matrix element reads

$$\Lambda_{\mathbf{k}}^{\nu\nu'} = U_{\mathbf{k}} \int \mathrm{d}^2\mathbf{r} (\varphi_{\mathbf{r}}^{\nu})^* \varphi_{\mathbf{r}}^{\nu'} 2i \sin(\frac{\mathbf{k}\cdot\mathbf{r}}{2}) \approx iU_{\mathbf{k}} \mathbf{d}_{\nu\nu'} \cdot \mathbf{k}/e_0 \ , \qquad (ka_x \ll 1) \tag{S2}$$

introducing the Coulomb interaction $U_{\mathbf{k}}$, the transition dipole moment $\mathbf{d}_{\nu\nu'} = e_0\langle\nu|\mathbf{r}|\nu'\rangle$ and the real-space exciton wave function $\varphi_{\mathbf{r}}^{\nu}$. Considering small momenta compared to the inverse exciton Bohr radius a_x^{-1}, the exciton-electron interaction can be expressed as

$$V_{x-e}(\mathbf{q}) = -\sum_{\mathbf{k},\nu\neq 0} \frac{U_{\mathbf{k}} U_{\mathbf{q-k}} (\mathbf{d}_{0\nu}\cdot\mathbf{k})(\mathbf{d}_{0\nu}^*\cdot(\mathbf{q}-\mathbf{k}))}{e_0^2(E_0^X - E_\nu^X)} \ , \qquad (ka_x \ll 1) \tag{S3}$$

which can be analytically evaluated in the long-wavelength limit ($q \to 0$):

$$\lim_{q\to 0} V_{x-e}(\mathbf{q}) = -\frac{A}{4\pi e_0^2} \int_0^{1/a_x} \mathrm{d}k k^3 (U_k)^2 \alpha \ , \alpha = \sum_{\nu\neq 0} \frac{|\mathbf{d}_{0\nu}|^2}{E_0^X - E_\nu^X} \tag{S4}$$

with A being the system area and α being the polarizability. The expression above can be evaluated for any form of the screened Coulomb interaction U_k. Note that we introduced a cut-off in the integral to be consistent with the approximation $ka_x \ll 1$. For the case of a pure 2D Coulomb interaction $U_k \sim 1/k$ we obtain

$$\lim_{q\to 0} V_{x-e}(\mathbf{q})|_{\mathrm{2D}} = -\frac{\alpha e_0^2}{32\pi\epsilon_0^2\epsilon_s^2 a_x^2 A} \ . \tag{S5}$$

We may also evaluate the $q \to 0$ limit of the exciton-electron interaction assuming a screened Coulomb interaction of the Keldysh form [2, 3] with $U_k = e_0^2/(2Ak\epsilon(k))$, $\epsilon(k) = \epsilon_0\epsilon_s(1 + r_0 k)$. Here, r_0 is the material-specific screening length, ϵ_s the dielectric constant of the surrounding substrate. We find then

$$\lim_{q\to 0} V_{x-e}(\mathbf{q})|_{\mathrm{Keldysh}} = -\frac{\alpha e_0^2}{16\pi\epsilon_0^2\epsilon_s^2 r_0^2 A} \left[\log(1 + \frac{r_0}{a_x}) - \frac{r_0}{r_0 + a_x}\right] \ . \tag{S6}$$

In the limit $r_0 \to 0$, Eq. (S5) and Eq. (S6) coincide as expected. The small momentum limit of the exciton-electron interaction enables us to find a quantitative estimate of the interaction strength. To get a material-specific value for the interaction, we evaluate Eq. (S6) and extract the polarizability from experiment [4]. The exciton Bohr radius a_x is microscopically computed from the Wannier equation and we find that $a_x \approx 1.5$ nm in the considered case of hBN-encapsulated $MoSe_2$ monolayers, resulting in an exciton-electron coupling strength of $v_{x-e} \approx -0.2$ eV nm^2 in the long wavelength limit (setting $A = 1$). The exciton-electron interaction is set to this constant value throughout our work, corresponding to performing a contact potential approximation of the full exciton-electron interaction in real space (Fourier-transform of Eq. (S1)). Finally, we note that, due to the (small) mass imbalance of electrons and holes in the $MoSe_2$, also first-order direct and exchange contributions to the exciton-electron interaction [5, 6] beyond the interaction matrix element considered in this work, should formally be taken into account. Within the long wave approximation, the corresponding direct interaction vanishes, and the dominant exchange interaction is assumed to be inefficient as we assume the Wigner crystal electrons and the charge constituents forming the exciton to be distinguishable.

II. LOCALIZATION LENGTH OF WIGNER ELECTRONS

We consider the electronic Hamilton operator

$$\hat{H}_e = \sum_i [-\frac{\hbar^2}{2m_e^*}\nabla^2_{\mathbf{r}_i} + \frac{1}{2}\sum_{j\neq i} U(\mathbf{r}_j - \mathbf{r}_i)] \ , \tag{S7}$$

where m_e^* is the effective carrier mass and $U(\mathbf{r}_i)$ is the Coulomb interaction. The total energy per lattice site i is then given by $E/N = \langle \hat{H}_e \rangle / N = K + W$ (with N being the total number of electrons) distinguishing between kinetic and potential contributions to the energy:

$$K = -\frac{\hbar^2}{2m_e^*}\int \mathrm{d}^2\mathbf{r}\psi_i^*(\mathbf{r})\nabla^2_{\mathbf{r}}\psi_i(\mathbf{r}) \ , W = \frac{1}{2}\int \mathrm{d}^2\mathbf{r}\mathrm{d}^2\mathbf{r}' U(\mathbf{r}-\mathbf{r}')|\psi_i(\mathbf{r})|^2 \sum_{j\neq i}|\psi_j(\mathbf{r}')|^2 \ , \tag{S8}$$

where we consider the Hartree contribution to the Coulomb interaction, which is the relevant contribution in the deep crystalline regime (away from Wigner crystal melting). Now, we perform the following Gaussian ansatz for the Wigner electron wave function:

$$\psi_i(\mathbf{r}) = \frac{1}{\sqrt{2\pi\xi^2}} \mathrm{e}^{-|\mathbf{r}-\mathbf{R}_i|^2/(4\xi^2)} \ , \tag{S9}$$

such that the Wigner crystal charge density reads $\rho(\mathbf{r}) = \sum_i |\psi_i(\mathbf{r})|^2$. Now, we would like to find the localization length ξ. This is done by minimizing the total energy per site and treating ξ as a variational parameter. Upon plugging the ansatz above into the kinetic energy in Eq. (S8) we find $K = \frac{\hbar^2}{4m_e^*\xi^2}$. Without loss of generality, we can fix the site $i = 0$ such that $\mathbf{R}_0 = \mathbf{0}$, as the ansatz is valid for any site i. For the potential energy, we find

$$\begin{aligned} W &= \frac{1}{2A(2\pi\xi^2)^2}\int \mathrm{d}^2\mathbf{r}\mathrm{d}^2\mathbf{r}' \sum_{j\neq 0,\mathbf{q}} U_{\mathbf{q}} \mathrm{e}^{i\mathbf{q}\cdot(\mathbf{r}-\mathbf{r}'+\mathbf{R}_j)} \mathrm{e}^{-\frac{r^2}{2\xi^2}} \mathrm{e}^{-\frac{r'^2}{2\xi^2}} \\ &= \frac{n_e}{2(2\pi\xi^2)^2}\int \mathrm{d}^2\mathbf{r}\mathrm{d}^2\mathbf{r}' \sum_{\mathbf{g}} U_{\mathbf{g}} \mathrm{e}^{i\mathbf{g}\cdot(\mathbf{r}-\mathbf{r}')} \mathrm{e}^{-\frac{r^2}{2\xi^2}} \mathrm{e}^{-\frac{r'^2}{2\xi^2}} - \frac{1}{2A(2\pi\xi^2)^2}\int \mathrm{d}^2\mathbf{r} d^2\mathbf{r}' \sum_{\mathbf{q}} U_{\mathbf{q}} \mathrm{e}^{i\mathbf{q}\cdot(\mathbf{r}-\mathbf{r}')} \mathrm{e}^{-\frac{r^2}{2\xi^2}} \mathrm{e}^{-\frac{r'^2}{2\xi^2}} \\ &= \frac{n_e}{2}\sum_{\mathbf{g}\neq 0} U_{\mathbf{g}} \mathrm{e}^{-|\mathbf{g}|^2\xi^2} - \frac{1}{4\pi}\int_0^\infty \mathrm{d}q q U_{\mathbf{q}} \mathrm{e}^{-\xi^2 q^2} \ , \end{aligned}$$

where we introduced the electron density $n_e = \frac{N}{A}$ and used $\sum_j \mathrm{e}^{i\mathbf{q}\cdot\mathbf{R}_j} = N\delta_{\mathbf{q},\mathbf{g}}$ with $\mathbf{g}$ being reciprocal lattice vectors of the Wigner lattice. Hence, the total energy per site can be expressed as

$$E = \frac{\hbar^2}{4m_e^*\xi^2} + \frac{n_e}{2}\sum_{\mathbf{g}\neq 0} U_{\mathbf{g}} \mathrm{e}^{-|\mathbf{g}|^2\xi^2} - \frac{1}{4\pi}\int_0^\infty \mathrm{d}q q U_{\mathbf{q}} \mathrm{e}^{-\xi^2 q^2} \ . \tag{S10}$$

The energy in Eq. (S10) can be minimized with respect to the variational parameter ξ for any lattice geometry and any form of the screened two-dimensional Coulomb interaction $U_{\mathbf{q}}$. Note that in the first term, $\mathbf{g} = 0$ is removed in order to cancel the contribution from the uniform positive background. In this work, we assume a Coulomb interaction of the Keldysh form [2, 3]. However, we can gain additional information about the density dependence of the localization length by considering a Coulomb interaction of the simple purely two-dimensional form $U_{\mathbf{q}} \sim \frac{1}{\epsilon q}$ with ϵ being the effective dielectric constant of the material. By minimizing the energy in this case, i.e., setting $\frac{dE}{d\xi} = 0$, we obtain the equation

$$-\frac{\hbar^2}{2m_e^*} - \frac{\xi^4}{\sqrt{3}a_W^2\epsilon}\sum_{\mathbf{g}\neq 0}|\mathbf{g}|\mathrm{e}^{-|\mathbf{g}|^2\xi^2} + \frac{\xi}{\sqrt{\pi}\epsilon} = 0 \ , \tag{S11}$$

introducing the Wigner lattice constant a_W, such that $n_e^{-1} = A_W = \frac{\sqrt{3}}{2}a_W^2$ assuming a triangular lattice. Numerically, it is found that the third term is much smaller than the second one containing a sum over all reciprocal lattice vectors $\mathbf{g} \neq 0$ and therefore this term can be neglected. By noting that $|\mathbf{g}| \propto \frac{1}{a_W}$ and assuming that $\xi \ll a_W$, it then follows that $\xi \propto a_W^{3/4} \propto n_e^{-3/8}$. Furthermore, by introducing the localization ratio $\xi/a_W \propto n_e^{1/8}$, we thereby expect the Wigner electrons to be better localized at lower densities.

III. DIAGONALIZATION OF EXCITON-WIGNER ELECTRON HAMILTONIAN

The mean-field exciton-Wigner electron Hamiltonian reads in the exciton picture

$$H = \sum_{\mathbf{Q}} E_{\mathbf{Q}} X^{\dagger}_{\mathbf{Q}} X_{\mathbf{Q}} + \sum_{\mathbf{q},\mathbf{Q}} V_{x-e}(\mathbf{q}) \rho_e(\mathbf{q}) X^{\dagger}_{\mathbf{Q}+\mathbf{q}} X_{\mathbf{Q}} \ , \tag{S12}$$

where the first term contains the free parabolic center-of-mass exciton dispersion $E_{\mathbf{Q}} = \frac{\hbar^2|\mathbf{Q}|^2}{2M}$ with $\mathbf{Q}$ being the center-of-mass momentum, and $M = m_e^* + m_h^*$ being the total exciton mass with m_e^* and m_h^* as electron and hole masses, respectively. The second term describes the mean-field interaction of excitons with Wigner crystal electrons, where the exciton-electron interaction $V_{x-e}(\mathbf{q})$ is weighted by the Wigner electron momentum charge density $\rho_e(\mathbf{q})$. The exciton-electron interaction can be approximated with a contact-like interaction in real space [1], such that $V_{x-e}(\mathbf{q}) = v_{x-e}$ (see Supplemental Section I). The Wigner electron charge density ρ_e is assumed to have a Gaussian density profile in momentum and real space, i.e, $\tilde{\rho}_e(\mathbf{r}) = \frac{1}{2\pi\xi^2}\sum_n \mathrm{e}^{-|\mathbf{r}-\mathbf{R}_n|^2/\xi^2}$ with $\mathbf{R}_n$ being real-space Wigner lattice vectors and ξ the localization length (discussed in Supplemental Section II). It then follows that the momentum space charge density reads

$$\rho_e(\mathbf{q}) = \frac{1}{A_W}\sum_{\mathbf{g}} \mathrm{e}^{-\xi^2|\mathbf{g}|^2/2}\delta_{\mathbf{q},\mathbf{g}} \ , \tag{S13}$$

where the Wigner unit cell area $A_W = \frac{\sqrt{3}}{2}a_W^2$ for a triangular lattice with a_W being the Wigner lattice constant. Importantly, $\rho_e(\mathbf{q}) = \sum_{\mathbf{g}} \bar{\rho}_e(\mathbf{g})\delta_{\mathbf{q},\mathbf{g}}$, i.e., periodic with respect to the reciprocal lattice vectors $\mathbf{g}$ of the Wigner crystal. Given the periodicity of the exciton-Wigner electron potential, we diagonalize the Hamiltonian in Eq. (S12) by performing a zone-folding, i.e., taking the center-of-mass momentum $\mathbf{Q} \to \mathbf{Q} + \mathbf{g}$ and restricting the summation over $\mathbf{Q}$ to the (mini)-Brillouin zone of the Wigner crystal. Furthermore, by introducing new operators $Y^{(\dagger)}_{\eta,\mathbf{Q}} = \sum_{\mathbf{g}} C^{(*)}_{\eta,\mathbf{g}}(\mathbf{Q}) X^{(\dagger)}_{\mathbf{Q}+\mathbf{g}}$, the Hamiltonian becomes diagonal such that

$$\tilde{H}_{x,0} = \sum_{\eta,\mathbf{Q}} \tilde{E}^{\eta}_{\mathbf{Q}} Y^{\dagger}_{\eta,\mathbf{Q}} Y_{\eta,\mathbf{Q}} \ , \tag{S14}$$

where $\tilde{E}^{\eta}_{\mathbf{Q}}$ is the renormalized exciton energy taking into account the exciton-Wigner electron potential. The renormalized exciton energies and the associated mixing coefficients $C_{\eta,\mathbf{g}}(\mathbf{Q})$ are obtained from solving the moiré eigenvalue problem

$$E_{\mathbf{Q}+\mathbf{g}} C_{\eta,\mathbf{g}}(\mathbf{Q}) + \sum_{\mathbf{g}'} W(\mathbf{g}-\mathbf{g}') C_{\eta,\mathbf{g}'}(\mathbf{Q}) = \tilde{E}^{\eta}_{\mathbf{Q}} C_{\eta,\mathbf{g}}(\mathbf{Q}) \ , \tag{S15}$$

with $W(\mathbf{g}) = V_{x-e}(\mathbf{g})\rho_e(\mathbf{g})$. The eigenvalue problem derived above is general and could be solved for any form of periodic interaction potential, W, and can be directly applied to excitons in a moiré potential induced by lattice mismatch or twisting in TMD-based heterostructures [7]. Note that we, for simplicity and given the low temperatures considered in this work, consider only the lowest-lying bright (KK) excitons and their interaction with electrons in a Wigner crystal in $MoSe_2$ monolayers.

IV. REAL-SPACE HAMILTONIAN AND LOCALIZATION OF EXCITONS

In real space, the mean-field exciton-electron Hamiltonian can be expressed as

$$H_x = \sum_i H_{x,i} = \sum_i [-\frac{\hbar^2\nabla^2_{\mathbf{r}_i}}{2M} + v_{x-e}\rho_i(\mathbf{r})] \ , \tag{S16}$$

where M is the total exciton mass (sum of electron and hole masses), v_{x-e} is the exciton-electron interaction, here assumed to be a contact interaction, and

$$\rho_i(\mathbf{r}) = \frac{1}{2\pi\xi^2}\mathrm{e}^{-|\mathbf{r}-\mathbf{R}_i|^2/\xi^2} \ , \tag{S17}$$

is the (Gaussian) real-space charge density of Wigner electrons. In the vicinity of the moiré lattice sites, i.e., $\mathbf{r} \approx \mathbf{R}_i$, we can approximate the mean-field interaction potential as a quantum harmonic oscillator potential and in particular (with $\mathbf{R}_0 = 0$), $\rho_0(\mathbf{r}) \approx \frac{1}{2\pi\xi^2}(1 - \frac{r^2}{\xi^2})$. In this case, it follows that the Wannier function associated with the lowest-lying exciton subband is Gaussian:

$$\phi_i(\mathbf{r}) = \frac{1}{\sqrt{\pi}a_x}\exp\left[-\frac{|\mathbf{r}-\mathbf{R}_i|^2}{2a_x^2}\right] , \tag{S18}$$

with the spatial extent a_x. By demanding that $H_{x,0}\phi_0(\mathbf{r}) = E\phi_0(\mathbf{r})$ for some constant energy E we find that

$$a_x = \left(\frac{\pi\hbar^2}{M|v_{x-e}|}\right)^{1/4}\xi , \tag{S19}$$

i.e., the spatial extension of the exciton wave function is directly proportional to the localization length of the Wigner electrons. Hence, by combining this result with the scaling of the localization ratio with respect to Wigner electron density, $\xi/a_W \propto n_e^{1/8}$, we have shown that the excitonic wave function becomes delocalized as n_e is increased. In other words, reducing the Wigner electron density is expected to result in a flattening of exciton bands. Notably, for excitons propagating in a moiré potential induced by a lattice-mismatch or a twist-angle in a TMD bilayer, it holds that $a_x/a_M \propto a_M^{-1/2} \propto n_M^{1/4}$ with a_M being the moiré period and n_M the moiré exciton density, i.e. a stronger density dependence is expected in such systems [8]. Given the weak exciton-electron potential ($|v_{x-e}| \approx 0.2$ eV nm^2), we find that the spatial extension of the exciton, a_x is slightly smaller and of similar order of magnitude as the Wigner lattice constant a_W for realistic Wigner electron densities ($n_e \sim 10^{11}$ cm^{-2}).

V. EXCITON-PHONON SCATTERING RATES

The periodic moiré potential that excitons feel and get trapped in due to the periodic Wigner lattice of electrons is analogous to the moiré potential induced by a lattice mismatch or by twisting in moiré materials [9]. In particular, the moiré eigenvalue problem used to obtain the renormalized exciton energies and mixing coefficients (Eq. (S15)) is general and can be applied to any form of periodic interaction potential. Therefore, the recently established theory for exciton-phonon scattering in moiré structures can be directly used also in our work. The exciton-phonon scattering rate in a moiré system is derived within a second-order Born-Markov approximation and reads [10]

$$\Gamma^{\eta'\eta}_{\mathbf{Q}'\mathbf{Q}} = \frac{2\pi}{\hbar}\sum_{\pm,j,\mathbf{g}} |\tilde{G}^{\eta'\eta}_{j,\mathbf{Q}\mathbf{Q}'\mathbf{g}}|^2 (\frac{1}{2} \pm \frac{1}{2} + n_{j,\mathbf{Q}-\mathbf{Q}'+\mathbf{g}})\delta(\tilde{E}^{\eta}_{\mathbf{Q}} - \tilde{E}^{\eta'}_{\mathbf{Q}'} \pm \hbar\omega_{j,\mathbf{Q}-\mathbf{Q}'+\mathbf{g}}) , \tag{S20}$$

where $\tilde{E}^{\eta}_{\mathbf{Q}}$ are the moiré exciton energies, η the exciton subband index, $\mathbf{Q}$ the center-of-mass momentum, and $\mathbf{g}$ the reciprocal lattice vectors of the Wigner lattice. Furthermore, $n_{j,\mathbf{q}}$ is the Bose distribution for phonons, $\hbar\omega_{j,\mathbf{q}}$ are the phonon energies and the moiré exciton-phonon matrix element reads [10, 11]

$$\tilde{G}^{\eta'\eta}_{j,\mathbf{Q}\mathbf{Q}'\mathbf{g}} = \sum_{\mathbf{g}',\tilde{\mathbf{g}}} G_{j,\mathbf{Q}-\mathbf{Q}'+\mathbf{g}} C^*_{\eta,\tilde{\mathbf{g}}}(\mathbf{Q}) C_{\eta',\mathbf{g}'}(\mathbf{Q}')\delta_{\mathbf{g},\mathbf{g}'-\tilde{\mathbf{g}}} , \tag{S21}$$

with the exciton-phonon matrix element $G_{j,\mathbf{q}}$ and the moiré mixing coefficients $C_{\eta,\mathbf{g}}(\mathbf{Q})$. The mixing coefficients and the corresponding moiré eigenenergies are obtained from zone-folding and diagonalizing the Hamiltonian in Eq. (1) in the main manuscript with the details provided in Supplemental Section III.

The relaxation time $\tau^{\eta}_{\mathbf{Q}}$ that enters the exciton diffusion coefficient and the transport calculation is extracted directly from the phonon-driven out-scattering rate as

$$(\tau^{\eta}_{\mathbf{Q}})^{-1} = \sum_{\eta',\mathbf{Q}'} \Gamma^{\eta'\eta}_{\mathbf{Q}'\mathbf{Q}} . \tag{S22}$$

The exciton-phonon coupling reads

$$G_{j,\mathbf{q}} = g^c_{j,\mathbf{q}} F(\beta\mathbf{q}) - g^v_{j,\mathbf{q}} F(-\alpha\mathbf{q}) , \tag{S23}$$

with the electron-phonon matrix elements $g^{\lambda}_{j,\mathbf{q}}$ and the form factors $F(\mathbf{q}) = \sum_{\mathbf{k}} \varphi^*_{\mathbf{k}+\mathbf{q}}\varphi_{\mathbf{k}}$ with $\varphi_{\mathbf{k}}$ being excitonic wave functions obtained from solving the Wannier equation [12, 13]. The mass ratio $\alpha = 1 - \beta = \frac{m_e^*}{m_e^*+m_h^*}$ is computed with

the effective masses m_e^* and m_h^* from *ab-initio* calculations [14]. We explicitly take into account intravalley exciton-phonon scattering involving longitudinal and acoustic phonon modes, $j = TA, LA$, relevant at the low temperatures considered in this manuscript [15]. Furthermore, the electron-phonon coupling matrix elements are treated within the deformation potential approximation [16]:

$$g_{j,\mathbf{q}}^{\lambda} = \sqrt{\frac{\hbar}{2\rho\omega_{j,\mathbf{q}}}} D_{j,\mathbf{q}}^{\lambda} \ , \tag{S24}$$

where ρ is the surface mass density of the TMD monolayer, A is the crystal area and $D_{j,\mathbf{q}}^{\lambda}$ is the deformation potential with λ being the band index and j the phonon mode. Note that the electron-phonon coupling for holes is related to that of valence band electrons as $D_{j,\mathbf{q}}^{h} = -D_{j,\mathbf{q}}^{v}$ and that $D_{j,\mathbf{q}}^{h}$ and $D_{j,\mathbf{q}}^{e}$ have the same sign, reflecting the non-polar nature of the interaction [17]. For long-wavelength acoustic phonons it holds that $D_{j,\mathbf{q}}^{\lambda} = \tilde{D}_j^{\lambda}\mathbf{q}$. Furthermore, the acoustic phonon frequencies read $\omega_{j,\mathbf{q}} = v_j|\mathbf{q}|$, where v_j is the speed of sound. The constant deformation potential $\tilde{D}_j^{\lambda}$ and sound speed v_j are obtained from DFPT calculations [16].

VI. EXCITON DIFFUSION IN VICINITY OF FERMI SEA OF ELECTRONS

The transport of excitons interacting with a Fermi sea of resident charge carriers can be studied using a Fermi-polaron approach as described in detail in Ref. [18]. The linewidth broadening due to exciton-electron scattering is given by [18]

$$\Gamma_{x-e}(n_e) = \frac{n_e(m_e^* + M)\hbar^2\pi}{Mm_e^*} \frac{\pi}{\ln^2(\delta/2E_t^b) + \frac{\pi^2}{4}} \ , \tag{S25}$$

where n_e is the free carrier density, m_e^* is the resident electron mass, M is the total exciton mass and E_t^b is the trion binding energy. The latter can be extracted from experiments on n-doped hBN-encapsulated $MoSe_2$ monolayers yielding $E_t^b = 27$ meV [19]. The effective electron and hole masses are extracted from *ab-initio* calculations [14] and are reported in Table I. Importantly, the exciton-electron scattering rate scales linearly with the carrier density or the Fermi energy. The parameter δ in Eq. (S25) is unrelated to the exciton-electron interaction and describes exciton broadening due to, e.g., the coupling with phonons. It relaxes the energy and momentum conservation for exciton-electron scattering. The diffusion coefficient is then given by

$$D = \frac{k_B T\hbar}{M[\delta + \Gamma_{x-e}(n_e)]} \ . \tag{S26}$$

In Fig. S1, we show the exciton diffusion coefficient of bright (KK) excitons in $MoSe_2$ using Eq. (S26) for different values of the phonon-induced broadening δ. We find a decrease of the diffusion coefficient with respect to density, reflecting the increased efficiency of the exciton-electron scattering at elevated densities. In this work, we set $\delta = 0.2$ meV such that the diffusion coefficient at the lowest considered carrier density ($n_e = 10^{11}$ cm^{-2}) coincides with the predicted diffusion coefficient for a free exciton ($D \approx 1$ cm^2/s). The opposite and qualitatively different density dependencies of exciton propagation in vicinity of an electron Wigner crystal and exciton transport in a Fermi sea hold also for a larger range of the broadening δ.

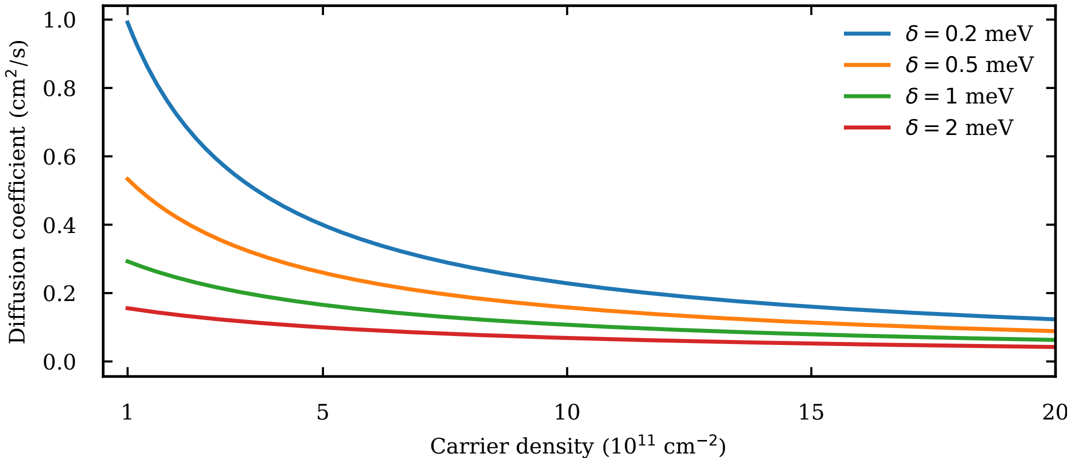

FIG. S1. Exciton diffusion in vicinity of Fermi sea of resident carriers as function of free carrier density for different values of the phonon-induced broadening δ.

VII. INPUT PARAMETERS

The material-specific parameters extracted from *ab initio* calculations are listed in Table I. We provide the effective mass of electrons and holes m^* (lowest-lying conduction band and highest-lying valence band at the K-point) in terms of the free electron mass m_0, the dielectric constant of hexagonal-boron nitride ϵ_s and the polarizability α. Furthermore, we provide the relevant phonon parameters for the calculation of exciton-phonon scattering rates including the speed of sound v and electron and hole deformation potentials $\tilde{D}^e$ and $\tilde{D}^h$ of longitudinal and transverse acoustic phonons.

Parameter	Value	Ref.
Eff. Electron (Hole) mass m^* $[m_0]$	0.5 (0.6)	[14]
Dielectr. hBN ϵ_s	4.5	[20]
Screening length r_0 [nm]	1	[21]
Polarizability α [eV nm^2/V^2]	6.532	[4]
Speed of sound v [cm/s]	$4.1{\cdot}10^5$	[16]
Electron (Hole) deformation potential $\tilde{D}$ [eV]	3.4 (2.8)	[16]

TABLE I. Material-specific parameters used for exciton transport calculations in hBN-encapsulated $MoSe_2$ monolayers. The phonon parameters are given for LA/TA phonons and are assumed to be the same for the longitudinal and transverse modes.

[1] D. K. Efimkin, E. K. Laird, J. Levinsen, M. M. Parish, and A. H. MacDonald, Electron-exciton interactions in the exciton-polaron problem, Phys. Rev. B **103**, 075417 (2021).
[2] L. Keldysh, Coulomb interaction in thin semiconductor and semimetal films, Sov. Phys. JETP **29**, 658 (1979).
[3] N. S. Rytova, The screened potential of a point charge in a thin film, Moscow University Physics Bulletin **3**, 18 (1967).
[4] L. Cavalcante, D. R. da Costa, G. Farias, D. Reichman, and A. Chaves, Stark shift of excitons and trions in two-dimensional materials, Phys. Rev. B **98**, 245309 (2018).
[5] G. Ramon, A. Mann, and E. Cohen, Theory of neutral and charged exciton scattering with electrons in semiconductor quantum wells, Physical Review B **67**, 045323 (2003).
[6] V. Shahnazaryan, I. Iorsh, I. A. Shelykh, and O. Kyriienko, Exciton-exciton interaction in transition-metal dichalcogenide monolayers, Phys. Rev. B **96**, 115409 (2017).
[7] S. Brem, C. Linderälv, P. Erhart, and E. Malic, Tunable phases of moiré excitons in van der Waals heterostructures, Nano Lett. **20**, 8534 (2020).

[8] F. Wu, T. Lovorn, E. Tutuc, and A. H. MacDonald, Hubbard model physics in transition metal dichalcogenide moiré bands, Phys. Rev. Lett. **121**, 026402 (2018).
[9] D. Huang, J. Choi, C.-K. Shih, and X. Li, Excitons in semiconductor moiré superlattices, Nat. Nanotechnol. **17**, 227 (2022).
[10] G. Meneghini, S. Brem, and E. Malic, Excitonic thermalization bottleneck in twisted tmd heterostructures, Nano Lett. **24**, 4505 (2024).
[11] G. Meneghini, S. Brem, and E. Malic, Spatiotemporal dynamics of moiré excitons in van der waals heterostructures, Nature Commun. **16**, 8557 (2025).
[12] S. Brem, J. Zipfel, M. Selig, A. Raja, L. Waldecker, J. D. Ziegler, T. Taniguchi, K. Watanabe, A. Chernikov, and E. Malic, Intrinsic lifetime of higher excitonic states in tungsten diselenide monolayers, Nanoscale **11**, 12381 (2019).
[13] M. Kira and S. W. Koch, Many-body correlations and excitonic effects in semiconductor spectroscopy, Progress in quantum electronics **30**, 155 (2006).
[14] A. Kormányos, G. Burkard, M. Gmitra, J. Fabian, V. Zólyomi, N. D. Drummond, and V. Fal'ko, k· p theory for two-dimensional transition metal dichalcogenide semiconductors, 2D Mater. **2**, 022001 (2015).
[15] M. Selig, G. Berghäuser, A. Raja, P. Nagler, C. Schüller, T. F. Heinz, T. Korn, A. Chernikov, E. Malic, and A. Knorr, Excitonic linewidth and coherence lifetime in monolayer transition metal dichalcogenides, Nature communications **7**, 13279 (2016).
[16] Z. Jin, X. Li, J. T. Mullen, and K. W. Kim, Intrinsic transport properties of electrons and holes in monolayer transition-metal dichalcogenides, Phys. Rev. B **90**, 045422 (2014).
[17] H. Peelaers and C. G. Van de Walle, Effects of strain on band structure and effective masses in MoS_2, Physical Review B—Condensed Matter and Materials Physics **86**, 241401 (2012).
[18] K. Wagner, Z. A. Iakovlev, J. D. Ziegler, M. Cuccu, T. Taniguchi, K. Watanabe, M. M. Glazov, and A. Chernikov, Diffusion of excitons in a two-dimensional Fermi sea of free charges, Nano Lett. **23**, 4708 (2023).
[19] J. Zipfel, K. Wagner, M. A. Semina, J. D. Ziegler, T. Taniguchi, K. Watanabe, M. M. Glazov, and A. Chernikov, Electron recoil effect in electrically tunable mo se 2 monolayers, Physical Review B **105**, 075311 (2022).
[20] R. Geick, C. H. Perry, and G. Rupprecht, Normal modes in hexagonal boron nitride, Phys. Rev. **146**, 543 (1966).
[21] D. Erkensten, S. Brem, R. Perea-Causin, and E. Malic, Stability of Wigner crystals and Mott insulators in twisted moiré structures, Phys. Rev. B **110**, 155132 (2024).
